# Supplementary material for: The Systems Biology Research Tool: evolvable open-source software
Source: BMC Syst Biol. 2008 Jun 29;2:55. doi: 10.1186/1752-0509-2-55 (PMC2446383; doi:10.1186/1752-0509-2-55)
Supplement: Additional file 1 — SBRT Archive. An archive of the current version of the Systems Biology Research Tool. [file 1752-0509-2-55-S1.zip › sbrt-1.4.0/doc/developers_guide/api/sbrt/shell/text/package-tree.html]

sbrt.shell.text Class Hierarchy


|  |  |  |
| --- | --- | --- |
| **Overview** \| **Package** \| Class \| Use \| **Tree** \| **Deprecated** \| **Index** \| **Help** | |  |
| **PREV**   **NEXT** | **FRAMES**    **NO FRAMES**     **All Classes** |  |


---


## Hierarchy For Package sbrt.shell.text

**Package Hierarchies:**: All Packages

---

## Class Hierarchy

- java.lang.Object
  - sbrt.shell.text.**AbstractLinkedHashSetFormat**<E> (implements sbrt.shell.text.LinkedHashSetFormat<E>)
    - sbrt.shell.text.**AmpersandSetFormat**<E>- sbrt.shell.text.**PipeSetFormat**<E>- sbrt.shell.text.**TabSetFormat**<E>- sbrt.shell.text.**WhitespaceSetFormat**<E>
    - sbrt.shell.text.**AbstractListFormat**<E> (implements sbrt.shell.text.ListFormat<E>)
      - sbrt.shell.text.**PipeListFormat**<E>- sbrt.shell.text.**TabListFormat**<E>- sbrt.shell.text.**WhitespaceListFormat**<E>
      - sbrt.shell.text.**AbstractMapEntryFormat**<K,V> (implements sbrt.shell.text.MapEntryFormat<K,V>)
        - sbrt.shell.text.**ColonMapEntryFormatV1**<K,V>- sbrt.shell.text.**EqualsMapEntryFormatV1**<K,V>
        - sbrt.shell.text.**AbstractMapFormat**<K,V> (implements sbrt.shell.text.MapFormat<K,V>)
          - sbrt.shell.text.**AppInputFileLineFormatVersion1** (implements sbrt.shell.text.AppInputFileLineFormat)- sbrt.shell.text.**ColonMapFormatV1**<K,V>- sbrt.shell.text.**EqualsMapFormatV1**<K,V>
          - sbrt.shell.text.**BoolRelFormatV1**<E> (implements sbrt.shell.text.SimpleFormat<T>)- sbrt.shell.text.**DoubleFormatV1** (implements sbrt.shell.text.DoubleFormat)- sbrt.shell.text.**DoubleFormatV2** (implements sbrt.shell.text.DoubleFormat)- sbrt.shell.text.**FileFormatFormat** (implements sbrt.shell.text.SimpleFormat<T>)- sbrt.shell.text.**IntegerFormatV1** (implements sbrt.shell.text.IntegerFormat)- sbrt.shell.text.**LongFormatV1** (implements sbrt.shell.text.LongFormat)- sbrt.shell.text.**NonEmptyStringFormat** (implements sbrt.shell.text.SimpleFormat<T>)- sbrt.shell.text.**PercentFormatVersion1** (implements sbrt.shell.text.PercentFormat)- sbrt.shell.text.**SimpleListFormat**<E> (implements sbrt.shell.text.SimpleFormat<T>)- sbrt.shell.text.**SimpleSetFormat**<E> (implements sbrt.shell.text.SimpleFormat<T>)- sbrt.shell.text.**SpecialChars**- sbrt.shell.text.**StringFormat** (implements sbrt.shell.text.SimpleFormat<T>)- java.lang.Throwable (implements java.io.Serializable)
                                    - java.lang.Exception
                                      - java.lang.RuntimeException
                                        - sbrt.shell.**ApplicationException**
                                          - sbrt.shell.text.**FormatException**
                                    - sbrt.shell.text.**TrueFalseFormat** (implements sbrt.shell.text.BooleanFormat)- sbrt.shell.text.**YesNoFormat** (implements sbrt.shell.text.BooleanFormat)

## Interface Hierarchy

- sbrt.shell.text.**Format**
  - sbrt.shell.text.**MapEntryFormat**<K,V>- sbrt.shell.text.**MapFormat**<K,V>
      - sbrt.shell.text.**AppInputFileLineFormat**
      - sbrt.shell.text.**SimpleFormat**<T> (also extends sbrt.shell.text.Formatter<T>, sbrt.shell.text.Parser<T>)
        - sbrt.shell.text.**BooleanFormat**- sbrt.shell.text.**CollectionFormat**<E>- sbrt.shell.text.**DoubleFormat**- sbrt.shell.text.**IntegerFormat**- sbrt.shell.text.**LongFormat**- sbrt.shell.text.**PercentFormat**
  - sbrt.shell.text.**Formatter**<T>
    - sbrt.shell.text.**LinkedHashSetFormat**<E> (also extends sbrt.shell.text.Parser<T>)- sbrt.shell.text.**ListFormat**<E> (also extends sbrt.shell.text.Parser<T>)- sbrt.shell.text.**SimpleFormat**<T> (also extends sbrt.shell.text.Format, sbrt.shell.text.Parser<T>)
          - sbrt.shell.text.**BooleanFormat**- sbrt.shell.text.**CollectionFormat**<E>- sbrt.shell.text.**DoubleFormat**- sbrt.shell.text.**IntegerFormat**- sbrt.shell.text.**LongFormat**- sbrt.shell.text.**PercentFormat**
    - sbrt.shell.text.**Parser**<T>
      - sbrt.shell.text.**LinkedHashSetFormat**<E> (also extends sbrt.shell.text.Formatter<T>)- sbrt.shell.text.**ListFormat**<E> (also extends sbrt.shell.text.Formatter<T>)- sbrt.shell.text.**SimpleFormat**<T> (also extends sbrt.shell.text.Format, sbrt.shell.text.Formatter<T>)
            - sbrt.shell.text.**BooleanFormat**- sbrt.shell.text.**CollectionFormat**<E>- sbrt.shell.text.**DoubleFormat**- sbrt.shell.text.**IntegerFormat**- sbrt.shell.text.**LongFormat**- sbrt.shell.text.**PercentFormat**

---


|  |  |  |
| --- | --- | --- |
| **Overview** \| **Package** \| Class \| Use \| **Tree** \| **Deprecated** \| **Index** \| **Help** | |  |
| **PREV**   **NEXT** | **FRAMES**    **NO FRAMES**     **All Classes** |  |


---
